# Characteristics of Housing Adaptation Recipients Under Japan’s Long-Term Care Insurance: A Cluster Analysis

**DOI:** 10.1093/geroni/igaf122.3282

**Published:** 2025-12-31

**Authors:** Satomi Kitamura, Tomoyuki Ota, Rumiko Tsuchiya-Ito

**Affiliations:** Institute for Health Economics and Policy, Association for Health Economics Research and Social Insurance and Welfare, Tokyo, Tokyo, Japan; The Hyogo Institute of Assistive Technology, Hyogo, Hyogo, Japan; Dia Foundation for Research on Ageing Societies, Tokyo, Tokyo, Japan

## Abstract

Housing adaptation is a key strategy for maintaining the functional ability of older adults with disabilities. This study aimed to characterize older adults aged ≥65 years who received housing adaptation under Japan’s long-term care (LTC) insurance in 2015. A k-means cluster analysis was conducted using anonymized medical and LTC claims data, and housing adaptation application data from Hachioji City, Tokyo. Clustering variables included age, sex, household income, LTC needs levels, basic activities of daily living, hospitalization history, comorbidities (10 diseases), housing type (detached house/apartment), and co-residence status (alone/spouse/others). In addition to these clustering variables, housing adaptation details and other LTC services were compared across clusters using Chi-square tests. Among 1,062 participants (median age 81[76–87], 57.1% female), three clusters were identified: 1) older adults with frailty (n = 420, 81[76–85]), 2) those with higher age and mild disabilities (n = 343, 88[84–91]), and 3) those with severe disabilities (n = 299, 75[71–79]). LTC needs levels increased from cluster 1 to 3. Cluster 1 had more joint diseases and fewer hospitalizations. Cluster 2 had more dementia, fractures, and non-spousal co-residing family. Cluster 3 included more males and recent hospitalizations. While housing adaptation details were similar across clusters (e.g., handrail installation: 84%, 82%, 86%; p = 0.44), LTC service utilization varied: cluster 1 used fewer services, cluster 2 relied more on home-visiting care and day-care services, and cluster 3 used more assistive product services (e.g., wheelchairs, electronic beds). These findings highlight the heterogeneity among housing adaptation recipients, suggesting the need for personalized planning.

